# Phenotype and Neuronal Cytotoxic Function of Glioblastoma Extracellular Vesicles

**DOI:** 10.3390/biomedicines10112718

**Published:** 2022-10-27

**Authors:** Wenbo Zhou, Daniel Lovasz, Zoë Zizzo, Qianbin He, Christina Coughlan, Robert G. Kowalski, Peter G. E. Kennedy, Arin N. Graner, Kevin O. Lillehei, D. Ryan Ormond, A. Samy Youssef, Michael W. Graner, Xiaoli Yu

**Affiliations:** 1 Department of Neurosurgery, University of Colorado Anschutz Medical Campus, Aurora, CO 80045, USA; wenbo.zhou@cuanschutz.edu (W.Z.); daniel.lovasz@cuanschutz.edu (D.L.); zoe.zizzo@cuanschutz.edu (Z.Z.); qianbin.he@cuanschutz.edu (Q.H.); robert.2.kowalski@cuanschutz.edu (R.G.K.); arin.graner@cuanschutz.edu (A.N.G.); kevin.lillehei@cuanschutz.edu (K.O.L.); david.ormond@cuanschutz.edu (D.R.O.); samy.youssef@cuanschutz.edu (A.S.Y.); michael.graner@cuanschutz.edu (M.W.G.); 2 Department of Neurology, University of Colorado Anschutz Medical Campus, Aurora, CO 80045, USA; christina.coughlan@cuanschutz.edu; 3 Institute of Infection, Immunity and Inflammation, University of Glasgow, Glasgow G12 8QQ, UK; peter.kennedy@glasgow.ac.uk

**Keywords:** extracellular vesicles, glioblastoma, meningioma, plasma, neurons, IgG, Fc, complement, C1q, apoptosis, neuroblastoma, cytotoxicity

## Abstract

Glioblastoma (GBM) is the most aggressive and lethal form of brain tumor. Extracellular vesicles (EVs) released by tumor cells play a critical role in cellular communication in the tumor microenvironment promoting tumor progression and invasion. We hypothesized that GBM EVs possess unique characteristics which exert effects on endogenous CNS cells including neurons, producing dose-dependent neuronal cytotoxicity. We purified EVs from the plasma of 20 GBM patients, 20 meningioma patients, and 21 healthy controls, and characterized EV phenotypes by electron microscopy, nanoparticle tracking analysis, protein concentration, and proteomics. We evaluated GBM EV functions by determining their cytotoxicity in primary neurons and the neuroblastoma cell line SH-SY5Y. In addition, we determined levels of IgG antibodies in the plasma in GBM (n = 82), MMA (n = 83), and controls (non-tumor CNS disorders and healthy donors, n = 50) with capture ELISA. We discovered that GBM plasma EVs are smaller in size and had no relationship between size and concentration. Importantly, GBM EVs purified from both plasma and tumor cell lines produced IgG-mediated, complement-dependent apoptosis and necrosis in primary human neurons, mouse brain slices, and neuroblastoma cells. The unique phenotype of GBM EVs may contribute to its neuronal cytotoxicity, providing insight into its role in tumor pathogenesis.

## 1. Introduction

Glioblastoma (aka, CNS5 WHO Grade 4 *IDH*-wild type astrocytoma; GBM), the most common type of malignant brain tumor in adults, is an aggressive cancer of glial origin that is universally fatal [1,2,3] and has an incidence rate of 3.21 per 100,000 with no truly effective therapy [4,5]. Current treatments, such as neurosurgical resection, radiotherapy, chemotherapy, and targeted immunotherapy have only improved 2 to 5-year survival rates slightly since 2005 for patients with GBM [6]. Meningiomas (MMA) are largely benign tumors formed from the meningeal layers of the brain [7,8]. However, 20% of MMA may display aggressive behavior [7] and develop early recurrences that require repeated surgeries, radiotherapy, or systemic treatments [9]. For these reasons, there is an urgent need to develop biofluid-based biomarkers for earlier diagnostic, disease progression, and drug response monitoring. There are several candidates of protein or microRNA-based biomarkers for GBM, such as glial fibrillary acidic protein (GFAP) [10,11,12], miR-340 [13], miR-185 [14], and miR-210 [15]; however, their values for prognostic prediction or disease progression monitoring remain unproven in clinical trials.

Extracellular vesicles (EVs) are membrane-enclosed nanovesicles released from multivesicular bodies (“exosomes”) or external budding of the plasma membrane (“microvesicles”) [16]. The cargos of exosomes and microvesicles, collectively referred to here as “extracellular vescicles”/EVs, derived from tumor cells contain tumor-specific proteins, DNAs, and RNAs, which could serve as potential diagnostic biomarkers. EVs are known to play integral roles in intercellular communication [17,18]. GBM cells communicate with surrounding cells by releasing EVs containing proteins, lipids, and RNA [19]. In addition, EVs have been shown to cross the blood–brain barrier bidirectionally [20], making it important to investigate the role of extracellular vesicles in the pathogenesis, diagnosis, and treatment of diseases such as brain tumors, Alzheimer’s disease, strokes, and cancer [21,22,23,24].

Brain tumors are known to have complex immune microenvironments [25,26] which harbor additional nonmalignant cells that contribute to the tumor’s progression [27,28]. There is a long history of antibody research in gliomas [29] to identify targetable tumor antigens. Numerous monoclonal antibody therapies, along with other forms of immunotherapy are currently under investigation as treatment options for individuals with GBM [30,31,32] and immunotherapies are also being investigated for individuals with high-grade MMA [25,33]. Questions nonetheless remain regarding the impacts of humoral responses in brain tumors.

Tumor-derived exosomes (EVs) were found to be bound to circulating immunoglobulins (IgG) in pancreatic adenocarcinoma (PDAC) and induced a dose-dependent inhibition of PDAC serum-mediated, complement-dependent cytotoxicity toward cancer cells [34]. IgG Fc domain mediates a wide range of effector functions including complement-dependent cytotoxicity (CDC). The binding of complement component 1q (C1q) triggers the activation of the complement cascade and leads to the formation of the membrane attack complex (MAC), which forms pores causing the lysis of target cells [35]. A deeper understanding of circulating IgG in GBM plasma-derived EVs would provide insight into the roles of EVs in disease pathogenesis.

We recently reported the identification of GBM EV-specific peptides which suggest that these EVs may possess unique features on the surface [36]. In the current study, we characterized the phenotypes of EVs derived from the plasma of patients with GBM, MMA, and controls. We demonstrate the unique features of GBM EVs and the presence of elevated levels of IgG antibodies (Fc) in GBM plasma. We further demonstrated that GBM EVs produced IgG-mediated, complement-dependent cytotoxicity in neurons, brain slices, and the neuroblastoma cell line SH-SY5Y. Our data support the important role of GBM EVs in disease pathogenesis, providing evidence supporting the interaction between tumor EVs and humoral immune response.

## 2. Materials and Methods

Human blood sample collection and preparation. This study was reviewed and approved by the Colorado Multiple Institutional Review Board (COMIRB) before sample collection. Written informed consent was obtained from each patient and healthy control subject by a Clinical Research Associate under the guise of COMIRB protocols #13-3007 and #18-1421. Blood samples of GBM, MMA, Metastatic Brain Tumors (Met), and other non-tumor CNS disorders were collected through the Tissue Bank at the Department of Neurosurgery, University of Colorado Anschutz Medical Campus (https://medschool.cuanschutz.edu/neurosurgery/research-and-innovation/services/nervous-system-biorepository (accessed on 2 September 2022)) [36]. Additional healthy control (HC) plasma was obtained from the Accelerated Cure Project (https://www.acceleratedcure.org, accessed on 2 September 2022). Briefly, the blood was collected in EDTA tubes (BD) and was centrifuged at 1500× *g* for 15 min at 4 °C. The plasma fraction was collected, aliquoted, and stored at −80 °C until use.

GBM and control cell lines. GBM tumor cell line and EV isolation, as well as controls, were the same as described [37].

### 2.1. Plasma Extracellular Vesicle Isolation

EV isolation with ExoEasy kit. We used the ExoEasy Maxi kit (Qiagen #76064, Hilden, Germany) for plasma EV isolation. About 3 mL of pooled plasma (mixed from 10 individual samples with equal volume) was filtered through a 0.8 µm filter (Sartorius #16592, Göttingen, Germany). The samples were mixed with 3 mL of XBP buffer and then loaded to the ExoEasy spin column for a spin at 500× *g* for 1 min. Then, 10 mL of XWP buffer was added to the column, followed by spin at 5000× *g* for 5 min. Finally, 400 mL of buffer XE was added to the column, followed by spin at 500× *g* for 5 min to collect the purified EVs. Purified EVs were stored at −80 °C.

EV isolation with ExoQuick^®^. ExoQuick^®^ kit (Systems Biosciences, Cat# EXOQ20A-1, Palo Alto, CA, USA) was also used for plasma EV isolation. Plasma samples were thawed and pre-cleared by centrifugation at 1500× *g* for 15 min at 4 °C. Briefly, 190 µL of plasma were mixed with 45 µL of ExoQuick ^®^ Exosome precipitation solution and incubated at 4 °C for 30 min, followed by centrifugation at 1500× *g* for 30 min at 4 °C. EV pellets were dissolved in 190 µL of sterile PBS and aliquoted samples were stored at −80 °C.

Cell line EV isolation using ultracentrifugation method. The protocol for EV isolation using the ultracentrifugation method has been described previously [37]. Briefly, the GBM tumor cell line and the control cell line conditioned media were collected after centrifugation (300× *g*, 5 min) to remove cells and debris. The media were further cleared through centrifugation at 17,000 rpm for 20 min in Sorvall Superspeed RC2-8 rotor. The supernatant was filtered through a 0.2 µm filter bottle (Thermo 566-0020, Waltham, MA, USA) and concentrated using a 100 kDa cut-off spin column (Sartorius VivaSpin 20, Göttingen, Germany) at 3000 rpm for 15 min in a Beckman centrifuge. The concentrated media were transferred to ultracentrifuge tubes (Beckman #C14277) for use in a Ti 70.1 rotor. The samples were centrifuged at 150,000× *g* for 2 h at 4 °C. The isolated EV pellets were re-suspended in 1 mL PBS and stored at -80ºC. This GBM cell line-derived EV material was termed GBM mEV. As a control, an epithelial cell line was derived from a healthy female donor. The control cell line-derived EV material was purified similarly to GBM mEV and termed Control mEV.

### 2.2. Characterization of Extracellular Vesicles

Negative staining of EV samples and transmission electron microscopy (TEM). The negative staining and EM observation of EV samples were performed at the Electron Microscopy Center of the University of Colorado School of Medicine. The EV specimen (5 µL) was dropped onto a 200–400 mesh carbon/formvar coated grid and allowed to adsorb to the formvar for one minute. The 2% uranyl acetate (10 µL) was placed on the grid for one minute. The sample was observed under a transmission electron microscope (FEI Tecnai G2 Biotwin TEM, Thermo Fisher, Waltham, MA, USA) at 80 KV with a magnification of 30,000 to 120,000x. The images were taken by the AMT camera system.

Nanoparticle Tracking Analysis. We used the Nanosight device (Malvern P analytical, Malvern, UK) for Nanoparticle Tracking Analysis (NTA) to evaluate EV sizes and concentrations. EVs were diluted 1:1000 in Milli-Q water (0.1 mm filtered) before reading. The experiments were repeated at least twice.

Protein concentration assay. Pierce Bicinchoninic acid (BCA) assay (Thermo Fisher, Waltham, MA, USA) was used. Samples were diluted in PBS and the assays were performed in 96-well plates. A standard curve was generated using BSA (0–2000 µg/mL). The plate was incubated at 37 °C for 30 min and read at 562 nm in a plate reader (Versa Max, Molecular Devices, San Jose, CA, USA).

### 2.3. Evaluating the Cytotoxicity of Cells Treated with GBM EVs

EV cytotoxicity assay in neuroblastoma cells. The neuroblastoma cells SH-SY5Y were obtained from ATCC (#CRL-2266). The cells were maintained in DMEM medium with 10% FBS and 1X Penicillin-Streptomycin and plated on 0.1% gelatin-coated 10-cm tissue culture plates. Cells were passaged once a week. The culture medium was removed and washed once with calcium/magnesium-free Hank’s buffer (Sigma, H8264), and 2 mL of trypsin/EDTA (0.05%/0.02%) solution was added to the cells and incubated at 37 °C for 5 min. Then, 10 mL of warm culture medium was added to dissociate the cells, followed by centrifugation at 500× *g* for 5 min. The cell pellets were suspended in 2 mL of medium and cell density was counted. Cells in passages 5–10 were used for cytotoxicity assays. Cells were plated at 20,000/cm^2^ in 96-well tissue culture plates (BD Falcon #353219). Cells were allowed to grow to 30–40% confluency before being used for EV treatment.

EV treatment components were mixed in a 96-well U-bottom sterile plate (Greiner Bio-One #6018-P113, Kremsmünster, Austria) plus 5% of Normal Human Serum as a source of complement (NHS, Complement Technology Inc., #NHS, Tyler, TX, USA), and the culture medium was added up to a total of 100 µL per well [36]. The cytotoxicity was measured by luminescence or fluorescence with the LIVE/DEAD™ Viability/Cytotoxicity kits (Invitrogen, Waltham, MA, USA) (1 µM Calcein AM, 2 µM Ethidium Homodimer-1), or Propidium Iodide (5 µg/mL), or Real-time-Glo Annexin V Apoptosis/Necrosis kits (Promega #JA1011, Madison, WI, USA). The plates were measured every 60 min with a plate reader (Biotek Synergy H4 hybrid reader, Winooski, VT, USA, software version Gen5 3.09) for 4 to 24 h.

EV cytotoxicity in normal human astrocytes. The normal human astrocytes (NHA) were obtained from ScienCell #1800 (passage 1) and cultured in Basal Medium (ScienCell #1801, Carlsbad, CA, USA), 2% of FBS (ScienCell #0010, Carlsbad, CA, USA), astrocytes growth supplement (ScienCell AGS, #1852, Carlsbad, CA, USA), and Penicillin/Streptomycin (1%). The astrocytes were passaged every 3–5 days when reaching 70% confluency. For the EV cytotoxicity test, the NHA was plated at 20,000 cells/cm^2^ in 96-well plates (BD Falcon #353219, Franklin Lakes, NJ, USA). The astrocytes were about 30% confluency at the time of the test. The GBM and Control EVs were mixed with 5% normal human serum and 1x apoptosis/necrosis reagents. The luminescence and fluorescence were measured for 0–4 h after EV treatment.

EV cytotoxicity in human primary neurons. The human primary neurons were obtained from ScienCell (#1520). The frozen vial (passage 0, 1 × 10^6^ cells) was thawed and plated on Poly-L-Lysine (2 µg/cm^2^) coated 96-well plate (BD Falcon #353219, Franklin Lakes, NJ, USA), at a density of 10,000 cells/cm^2^. The culture medium consisted of 98% Neuronal Medium (ScienCell #1521, Carlsbad, CA, USA), 1% Neuronal Growth Supplement (NGS, ScienCell #1562, Carlsbad, CA, USA), and 1X Penicillin/Streptomycin. The medium was changed every 2 to 3 days. The neurons were treated on day 8 with GBM and control EVs, 5% Normal Human Serum, and 1x Live/Dead assays (final 1 µM Calcein AM/2 µM Ethidium Homodimer1).

EV cytotoxicity in newborn mouse brain slices. The animal protocol was reviewed and approved by the University of Colorado Institutional Animal Care and Use Committee (IACUC). The pregnant Balb/c mice were purchased from Charles River Laboratories (Wilmington, MA, USA). Newborn mice (P0-P1) were anesthetized by hypothermia and euthanized by decapitation. Brain tissues were cut into thin slices approximately 1 mm in thickness and 2 mm round pieces with a tissue biopsy puncher (Zivic Instruments Inc., PUN2000, Pittsburgh, PA, USA). The tissue slices were maintained in a 96-well plate overnight in neural basal A medium supplemented with 10 µg/mL bFGF and 10 µg/mL EGF. GBM cell line medium EV (mEV) or control cell line mEV (20 µg/well each), 5% NHS, and 1x Apoptosis/Necrosis detection reagents were incubated with the brain tissues. Neuronal apoptosis was measured by luminescence in a plate reader (Biotek, Winooski, VT, USA) for 5 h post-treatment.

IdeS digestion and C1q antibody to inhibit EV cytotoxicity. EVs were digested with FabRICATOR (IdeS, Genovis #A0-FR1-008, Lund, Sweden). Five U (1U/µL) of IdeS was used to digest 30 µg of EVs in PBS (total of 30 µL) at 37 °C for 30 min. To test if inhibiting complement complex formation can block EV-induced cytotoxicity, a rabbit anti-C1q antibody (DAKO, A0136, Glostrup, Denmark) was added to the cells during the EV treatment (20 µg/well) in SH-SY5Y cells. The cytotoxicity was measured by apoptosis/necrosis assays (Promega, JA1011, Madison, WI, USA).

### 2.4. ELISA Determining Total IgG Antibodies in Plasma and Extracellular Vesicles

For all ELISA assays, MaxiSorp ELISA plates (Thermo #436110, Waltham, MA, USA) were used. NeutrAvidin-HRP (Thermo #31030, Waltham, MA, USA) and TMB substrate (SeraCare, Milford, MA, USA) were applied for detection. The Human IgG standard from Invitrogen human IgG ELISA Kit (Thermo Fisher, 991000, Waltham, MA, USA) was included in all experiments.

ELISA detecting Fc IgG antibodies with capture anti-Fc IgG in plasma and EVs. ELISA plates were coated in duplicates with goat anti-human IgG-Fc antibody (Rockland #609-1103, Pearl River, NY, USA) at 10 µg/mL (100 µL/well) overnight at 4 °C. The plates were blocked with 3% BSA for 2–5 h. Plasma (1:240 dilution in PBS) or EVs (25 µg/mL) were incubated overnight at 4 °C, followed by detection with biotinylated goat anti-human IgG (H + L) antibody (1:10,000, Vector Lab #BA3000, Burlingame, CA, USA), and NeutrAvidin-HRP (1:20,000). TBM substrate was applied followed by the addition of 0.1N HCl to stop the reaction. The plates were read at 450 nm by a microplate reader (BioTek Synergy plate reader, Winooski, VT, USA).

ELISA detecting Fc IgG antibodies with Fc binding peptides. Fc III, a 13-mer IgG-Fc domain binding peptide [38] was coated as a capture reagent. Synthetic Fc-III peptides (DCAWHLGELVWCT, New England Peptide, Gardner, MA, USA) at 100 µg/mL in 0.1M NaHCO3, pH 9.4 were coated onto ELISA wells, followed by the addition of plasma (1:240 dilution in PBS) or EVs (25 µg/mL). Bound IgG antibodies were detected with biotinylated goat anti-human IgG (H + L) antibody as described above.

ELISA detecting IgG1. Mouse anti-IgG1 antibodies (Sigma #I2513, Saint Louis, MO, USA) at a final concentration of 10 µg/mL were used to coat the plate. Plasma dilution (1:2000) or EVs (25 µg/mL) was added, followed by detection with biotinylated goat anti-human IgG-Fc antibody (1:10,000, Rockland #609-1603, Pearl River, NY, USA) and NeutrAvidin-HRP as described above.

### 2.5. Protein Digestion and Mass Spectrometry Analysis

Purification of plasma IgG using a Melon™ column. The Melon™ Gel IgG Spin Purification Kit (Thermo Fisher #45206, Waltham, MA, USA) was used to purify IgG from plasma. Briefly, 20 µL plasma was diluted at 1:10 and mixed with 100 µL of Melon™ gel slurry. Purified IgG was determined by BCA assay and stored at −80 °C.

Mass Spectrometry Proteomic analysis. Mass spectrometry-based proteomic analyses were performed at the Proteomics Core Facility of the University of Colorado Cancer Center. The purified IgG and EV samples from the ExoQuick kit were digested with trypsin, followed by peptide extraction using an S-Trap filter. Samples were analyzed on an Orbitrap Fusion mass spectrometer (Thermo Fisher Scientific) coupled to an Easy-nLC 1200 system (Thermo Fisher Scientific, Waltham, MA, USA) through a nanoelectrospray ion source. Peptides were separated on a self-made C18 analytical column (100 µm internal diameter, ×20 cm length) packed with 2.7 µm Cortecs particles. After equilibration with 3 µL 5% acetonitrile 0.1% formic acid, the peptides were separated by a 120 min linear gradient from 6% to 38% acetonitrile with 0.1% formic acid at 400 nL/min. LC mobile phase solvents and sample dilutions used 0.1% formic acid in water (Buffer A) and 0.1% formic acid in 80% acetonitrile (Buffer B) (Optima™ LC/MS, Fisher Scientific, Pittsburgh, PA, USA). Data acquisition was performed using the instrument supplied Xcalibur™ (version 4.1) software. Survey scans covering the mass range of 350–1800 were performed in the Orbitrap by scanning from *m*/*z* 300–1800 with a resolution of 120,000 (at *m*/*z* 200), an S-Lens RF Level of 30%, a maximum injection time of 50 ms, and an automatic gain control (AGC) target value of 4e5. For MS2 scan triggering, monoisotopic precursor selection was enabled, charge state filtering was limited to 2–7, an intensity threshold of 2e4 was employed, and dynamic exclusion of previously selected masses was enabled for 45 s with a tolerance of 10 ppm. MS2 scans were acquired in the Orbitrap mode with a maximum injection time of 35 ms, quadrupole isolation, an isolation window of 1.6 *m*/*z*, HCD collision energy of 30%, and an AGC target value of 5e4.

MS/MS spectra were extracted from raw data files and converted into mgf files using Proteome Discoverer Software (ver. 2.1.0.62). These mgf files were then independently searched against the human database using an in-house Mascot server (Version 2.6, Matrix Science, Chicago, IL, USA). Mass tolerances were ±10 ppm for MS peaks, and ±25 ppm for MS/MS fragment ions. Trypsin specificity was used allowing for 1 missed cleavage. Met oxidation, protein N-terminal acetylation, and peptide N-terminal pyroglutamic acid formation was allowed as variable modifications. Scaffold (version 4.8, Proteome Software, Portland, OR, USA) was used to validate MS/MS-based peptide and protein identifications. Peptide identifications were accepted if they could be established at greater than 95.0% probability as specified by the Peptide Prophet algorithm. Protein identifications were accepted if they could be established at greater than 99.0% probability and contained at least two identified unique peptides.

The partial least squares-discriminant analysis (PLS-DA), volcano graphs, and heatmaps were performed using the MetaboAnalyst 5.0 online platform with sum and range scaling normalizations. https://www.metaboanalyst.ca/MetaboAnalyst/ModuleView.xhtml (accessed on 2 September 2022).

Proteomic data were further analyzed using QIAGEN Ingenuity Pathway Analysis (IPA; QIAGEN Inc., Hilden, Germany. https://digitalinsights.qiagen.com/IPA (accessed on 2 September 2022).

### 2.6. Data/Statistical Analysis

One-way ANOVA analyses with Tukey’s correction for multiple comparisons were performed to determine if there were any statistically significant differences between treatments and groups. The X-Y data set correlation analysis was performed to compute Pearson correlation coefficients using GraphPad Prism 8.0 software and the regression lines were plotted. Data are presented as Mean ± SEM (or SD).

## 3. Results

### 3.1. Glioblastoma Plasma-Derived Extracellular Vesicles Possess Unique Phenotypes

**Figure 1:**
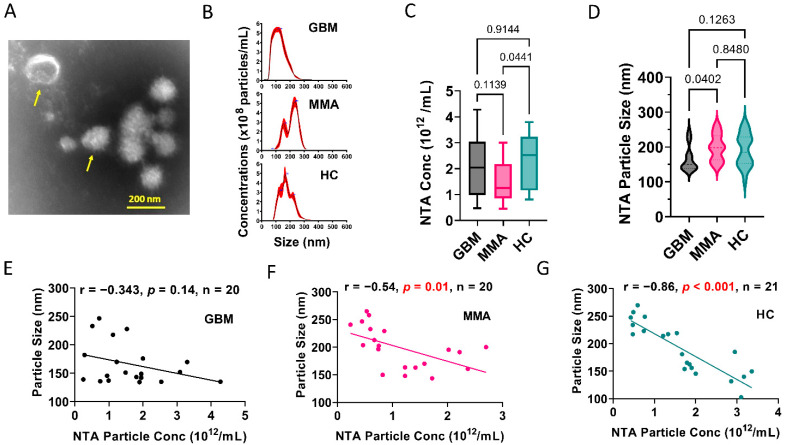
Glioblastoma plasma-derived extracellular vesicles possess unique phenotypes. (**A**). Representative transmission electron microscopic image of EVs derived from pooled GBM plasma. Yellow arrows show typical EVs. The mean size of the GBM EVs is 100–200 nm. (**B**). Nanoparticle Tracking Analysis (NTA) showing representative graphs for EVs purified from plasma of patients with GBM and MMA, and healthy controls (HC). (**C**). Summary of the number of EVs in the plasma of GBM, MMA, and HC, as determined by NTA. MMA EVs showed lower numbers of EVs (n = 20) compared to HC EVs (n = 21). (**D**). GBM plasma EVs are significantly smaller (n = 20) compared to MMA EVs (n = 20). The mean size of GBM EV is 166 nm. (**E**–**G**). Correlation analysis between NTA particle concentrations and particle sizes. (**E**). No correlation between concentration and size was observed for GBM EVs (r = −0.343, *p* = 0.14, n = 20). However, significantly correlated results were seen in MMA EVs (r = −0.54, *p* = 0.01, n = 20) (**F**) and HC EVs (r = −0.86, *p* < 0.001, n = 21) (**G**).

We previously showed that glioblastoma cell line-derived extracellular vesicles drive normal astrocytes toward a tumor-enhancing phenotype [37]. To further investigate the characteristics of GBM EVs, we purified plasma EVs using the ExoQuick kit from 20 patients with GBM, 20 MMA, and 21 healthy controls (Patient demographics in Table S1). We examined the EV morphology under transmission electron microscopy (TEM). A representative image of GBM plasma EVs showed several EVs with a size range of 100–200 nm (Figure 1A). We further characterized the extracellular vesicles using Nanoparticle tracking analysis (NTA) (Figure 1B–D). Figure 1B shows the representative NTA tracks for each group. On average, MMA EVs showed lower levels of particle concentrations (n = 20) compared to HC EVs (n = 21) (Figure 1C). However, GBM plasma EVs had a significantly smaller size of about 166 nm (n = 20) compared to MMA EVs (n = 20) (Figure 1D). To see if there was any correlation between EV particle size and concentrations, we performed a correlations analysis. In GBM EVs, no correlation between concentration and size was observed (r = −0.343, *p* = 0.14, n = 20) (Figure 1E). However, significantly correlated results were seen in MMA EVs (r = −0.54, *p* = 0.01, n = 20) (Figure 1F) and HC EVs (r = −0.86, *p* < 0.001, n = 21) (Figure 1G). These data demonstrated a unique feature of plasma EVs from patients with GBM compared to plasma EVs from patients with MMA and HC.

### 3.2. GBM EVs Derived from Both Plasma and Tumor Cell Lines Induced IgG-Mediated, Complement-Dependent Cytotoxicity in Neurons

**Figure 2:**
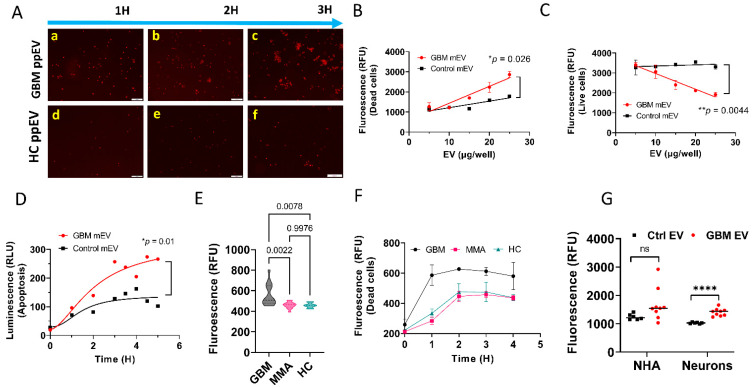
GBM extracellular vesicles derived from both plasma and tumor cell lines produced cytotoxicity in neurons and brain tissues. (**A**). Fluorescent microscopy images showing that GBM plasma EVs produced cell death in neuroblastoma cells in a time-dependent manner. SH-SY5Y cells (30–40% confluency) were incubated with GBM plasma and control EVs (50 µg/mL), 5% Normal Human Serum as a complement source, and propidium iodide. GBM pooled plasma EVs (GMB ppEV) produced large numbers of dead cells (red fluorescence) in a time-dependent manner (**a**–**c**) compared with control plasma EV (HC ppEV) treated cells (**d**–**f**). Scale bar = 100 nm. (**B**,**C**). Neuronal death caused by GBM EVs is dose-dependent. (**B**). A significantly higher number of dead cells were observed in GBM tumor cell line medium EVs (mEV) treated cells compared with controls in a dose-dependent manner (* *p* = 0.026). (**C**). Significantly lower number of live cells was detected in GBM mEV-treated cells compared with controls in a dose-dependent manner (** *p* = 0.0044). (**D**). GBM cell line EVs produced apoptosis in mouse brain tissues. P1 mouse brain slices (2 mm diameter) were incubated with GBM mEV and apoptosis was measured by luminescence. GBM mEVs produced significantly higher levels of apoptosis compared to controls (* *p* = 0.01). (**E**,**F**). GBM plasma EVs induced higher levels of cytotoxicity in SH-SY5Y cells. Plasma EVs were purified by ExoQuick and incubated with neuroblastoma cells. Cell death was measured by propidium iodide. (**E**). GBM plasma EVs produced significantly high levels of cytotoxicity compared to MMA (*p* = 0.002) and HC EVs (*p* = 0.008). n = 14 for GBM and MMA, n = 8 for HC. (**F**). Time-dependent cytotoxicity by GBM EVs. (**G**). GBM medium EVs did not induce significant cytotoxicity to normal human astrocytes (NHA) as compared to Control medium EVs. GBM and Control mEVs, used to treat the neurons and astrocytes, were purified by ultracentrifugation. Cell death was measured by propidium iodide. The results showed no significant cytotoxicity in astrocytes treated with GBM mEV versus Control mEV treated cells (n = 9 for GBM mEV, n = 7 for Control mEV, ns, not significant, **** *p* > 0.1).

To evaluate whether GBM EVs induce cytotoxicity in human neurons, we used neuroblastoma SH-SY5Y cells as surrogates for neurons. We first incubated GBM plasma EVs plus normal human serum (as a complement source) with SH-SY5Y cells in the presence of propidium iodide (PI) for 1–3 h. PI binds to double-stranded DNA but is excluded from cells with intact plasma membranes. The DNA in dying or dead cells was stained by PI which emitted red fluorescence. Figure 2A are fluorescent microscopy images showing that GBM plasma EVs purified by the ExoEasy kit can produce cytotoxicity in neuroblastoma cells in a time-dependent manner (a–c). In contrast, Control plasma EVs, also purified by the ExoEasy kit, did not show significant cell death (d–f).

To further confirm GBM EV cytotoxic effects, we treated primary human neurons (ScienCell, Carlsbad, CA, USA) with EVs, purified by ultracentrifugation [37], from a GBM or a control (epithelial cells of a healthy female) cell line. We quantified the neuronal cytotoxicity using the LIVE/DEAD™ Viability/Cytotoxicity Kit (Thermo Fisher, Waltham, MA, USA). A significantly higher number of dead neurons were observed in neurons treated with GBM tumor cell line EVs (mEV) versus the Control EVs. This effect was dose-dependent (Figure 2B, *p* = 0.026). As expected, a significantly reduced number of live cells were detected in GBM mEV-treated neurons compared with Controls. This effect was also dose-dependent (Figure 2C, *p* = 0.0044).

We next tested if GBM mEVs, purified by the ultracentrifugation method, have cytotoxic effects on brain tissue. We incubated GBM mEVs with neonatal mouse brain slices. We used the Real-time-Glo™ Annexin V Apoptosis and Necrosis Assay (Promega, Madison, WI, USA) to evaluate EV cytotoxicity. P1 mouse brain slices (2mm diameter) were incubated with GBM mEVs and apoptosis was measured by luminescence. GBM mEVs produced significantly higher levels of apoptosis compared to Control mEVs (Figure 2D, *p* = 0.01). To further validate whether the cytotoxicity was specific to GBM-derived EVs, we compared neuronal cytotoxicity (in SH-SY5Y) caused by GBM, MMA, and HC plasma-derived EVs, all purified from individual plasma using the ExoQuick kit. We demonstrated that GBM plasma EVs produced significantly higher levels of cytotoxicity (Figure 2E) compared to MMA (*p* = 0.002) and HC EVs (*p* = 0.008) (n = 14 for GBM and MMA, n = 8 for HC). Figure 2F shows time-dependent cytotoxicity by GBM EVs compared to MMA and HC EVs. As a comparison, we also tested whether GBM EVs have any cytotoxicity to astrocytes. Using the same conditions on neurons, we found that GBM mEV (purified by Ultracentrifugation) did not induce significant cytotoxicity to normal human astrocytes as compared to control mEVs (Figure 2G, *p* > 0.1). The results indicate that GBM mEV only caused cytotoxicity to neurons but not to astrocytes.

### 3.3. GBM EV-Induced Neuronal Cytotoxicity Requires IgG Antibodies and C1q

**Figure 3:**
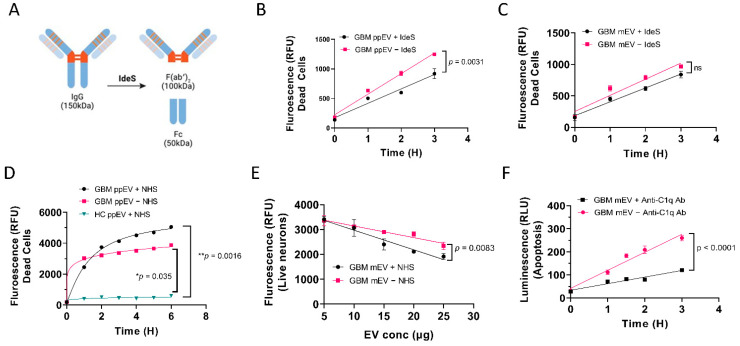
Reduced levels of GBM EV-induced neuronal cytotoxicity were observed by IgG digestion and complement elimination. (**A**). Schematic drawing showing digestion of IgG with the highly specific IgG cleaving enzyme IdeS. B-C. Reduced levels of cell death were observed in SH-SY5Y cells with IdeS-digested GBM EVs. GBM EVs were digested with IdeS before incubating with cells. (**B**). Significantly lower levels of cytotoxicity were found in cells treated with IdeS digested GBM pooled plasma EVs (ppEV) compared to undigested EVs (*p* = 0.0031). (**C**). Although not significant, a similar trend was seen when treating cells with IdeS digested GBM cell line medium-derived EVs (mEV) compared to undigested EVs. (**D**). Normal human serum (NHS) enhanced GBM plasma EVs induced cytotoxicity in SH-SY5Y cells. GBM ppEVs (5 µg/well) were added to cells with or without 5% NHS and the cytotoxicity was measured by green-fluorescent necrosis dye. NHS increased GBM ppEV cytotoxicity when compared to GBM ppEV treatment without NHS. However, without NHS, GBM ppEVs nonetheless induced elevated cytotoxicity compared to HC ppEVs. (**E**). Live cell counts post EV treatment. Human primary neurons were treated with GBM mEVs with or without 5% NHS. The cytotoxicity was measured using the live/dead assay (1 µM Calcein AM/2 µM Ethidium Homodimer1). Increasing GBM EV concentration produced fewer live cells, and NHS treatment enhanced the GBM EV cytotoxicity compared to samples without NHS (*p* = 0.0083). (**F**). Blocking of complement component C1q with anti-C1q antibody inhibits GBM EV-induced apoptosis. Rabbit anti-C1q antibodies (4 µg/well) were incubated with cells during GBM EV treatment. C1q antibody produced significantly lower levels of apoptosis in a time-dependent manner (*p* < 0.0001).

We consistently noticed that adding normal human serum (NHS) as a complement source enhanced GBM EV cytotoxicity, suggesting that GBM EVs may induce complement activation via the classical pathway that involves IgG antibodies. To test if IgG antibodies are required for GBM EV-induced cytotoxicity, we digested GBM EVs with a highly specific IgG-cleaving enzyme derived from Streptococcus pyogenes IdeS [39]. IdeS specifically digests the IgG hinge region and produces the F(ab’)2 and Fc fragments (Figure 3A). Significantly lower levels of cytotoxicity were found in IdeS digested, GBM pooled plasma EV (ppEV) treated cells, compared to undigested EV treated (*p* = 0.0031, Figure 3B). Although not as significant, a similar trend was seen in IdeS digested GBM mEV treated cells compared to undigested EVs (Figure 3C). These data suggest that the cytotoxicity of GBM plasma EVs was mediated by IgG antibodies.

We further investigated the role of the complement complex in the GBM EV cytotoxicity. We compared GBM EV treatment with or without NHS in human neuronal cells. NHS increased GBM ppEV cytotoxicity compared to GBM ppEV treatment without NHS. However, even without NHS, GBM ppEVs produced elevated cytotoxicity compared to HC ppEVs, indicating that complement, present in the NHS, may assemble on the surface of GBM EVs to induce cytotoxicity (Figure 3D). Consequently, when more cells died, there were fewer live cells. We also measured the live cells as a complimentary validation for GBM EV-induced cytotoxicity. Figure 3E shows that increasing GBM EV doses resulted in fewer live cells. In addition, the inclusion of NHS enhanced the GBM EV cytotoxicity compared to samples without NHS (*p* = 0.0083, Figure 3E). To determine if complement C1q is required for GBM EV cytotoxicity, we tested whether adding the anti-C1q antibody to the treatment mixture can block the cytotoxicity. Rabbit anti-C1q antibodies (4 µg/well) were incubated with cells during GBM EV treatment. The addition of the C1q antibody significantly reduced levels of apoptosis in a time-dependent manner (*p* < 0.0001, Figure 3F). Collectively, these results indicated cytotoxicity of GBM plasma or cell line-derived EVs was largely IgG antibody-mediated and complement-dependent.

### 3.4. Higher Levels of Total IgG (Fc) and IgG1 Antibodies Were Present in GBM Plasma, but Not in Plasma-Derived EVs from GBM Patients

As GBM EVs exhibited what seemed to be IgG antibody-mediated cytotoxicity, we asked if higher levels of IgG Fc fragments were present in GBM plasma and GBM plasma EVs.

**Figure 4:**
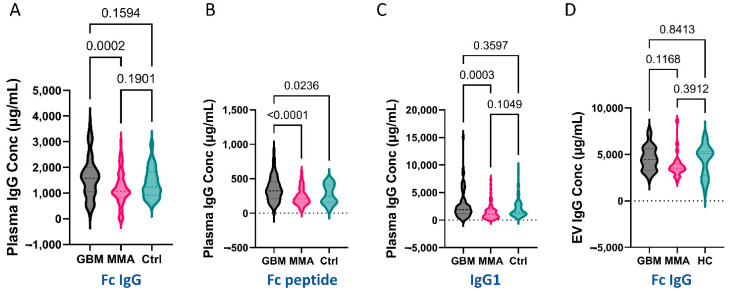
Higher levels of IgG antibodies were detected in GBM plasma with a similar trend in GBM plasma EVs. For plasma ELISA, a total of 82 GBM, 83 MMA, and 50 control plasmas were used. (**A**,**B**). Significantly higher levels of total Fc IgG antibodies were detected in GBM plasma. The 96-well MaxiSorp ELISA plates were coated with capture antibodies (10 µg/mL goat anti-human IgG Fc antibody, (**A**), or 100 µg/mL of synthetic Fc-III-peptide (**B**) overnight at 4 °C. Fc IgG antibodies in diluted plasma were detected by biotinylated goat anti-human IgG (H + L) antibody followed by the addition of NeutrAvidin-HRP and TMB. (**A**). Higher Fc-IgG antibodies were detected in GBM plasma compared to MMA plasma (*p* = 0.0002). (**B**). GBM plasma had significantly higher levels of Fc-IgG captured by Fc peptides compared to both MMA (*p* < 0.0001) and controls (*p* = 0.02). (**C**)**.** Higher levels of IgG1 in GBM plasma compared to MMA. Mouse anti-human IgG1 antibodies were coated on ELISA plates overnight followed by the addition of plasma (1:2000). Biotinylated goat anti-human IgG-Fc antibody and NeutrAvidin-HRP were used for detection. IgG1 levels in GBM plasma were significantly higher compared to MMA plasma (*p* = 0.0003), but not significantly different to controls. (**D**). No significant difference was detected in the Fc IgG levels among GBM, MMA, and HC EVs (GBM = 26, MMA = 26, HC = 20).

We developed an in-house ELISA to measure plasma levels of total IgG Fc using goat anti-human IgG Fc antibody. We also utilized Fc-III-peptides as a coating reagent. Bound IgG antibodies were detected with biotinylated goat anti-human IgG (H + L) followed by detection with NeutrAvidin-HRP. We demonstrated that GBM plasma had significantly higher IgG levels than MMA plasma, but not significantly higher than controls (*p* = 0.0002 GBM vs. MMA, Figure 4A). In the Fc-III peptide-coated ELISA, we found that GBM plasma had a significantly higher level of Fc-IgG compared to both MMA and control plasma (*p* < 0.0001 for MMA; *p* = 0.0236 for control, Figure 4B). These data indicate that the Fc peptides are a better choice for evaluating levels of IgG. In addition, we showed that the IgG1 levels in GBM plasma were significantly higher than that in MMA (*p* = 0.0003, Figure 4C). Furthermore, we measured total IgG and IgG1 levels in the EVs purified from the GBM, MMA, and HC plasma. EVs were directly used for the assays without lysis. Unlike the plasma IgG levels, we did not detect significant differences in the levels of total IgG among GBM, MMA, and HC EVs in the anti-Fc coated ELISA (Figure 4D) or Fc-peptide coated or IgG1-coated ELISA (data not shown).

### 3.5. Proteomics Analysis Revealed Unique Protein Profiles for GBM EVs

**Figure 5:**
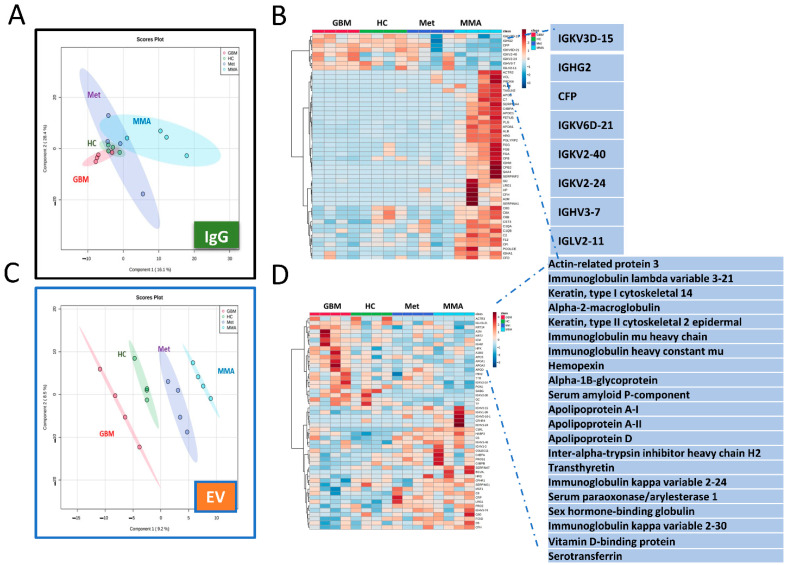
Mass spectrometry proteomic analysis of EVs and purified IgG antibodies showing unique clusters of proteins in GBM. (**A**,**B**). Proteomic analysis for IgG antibodies. (**A**). Principal Component Analysis (PCA) showed distinctions among the four groups of samples including GBM, MMA, metastatic brain tumors (Met), and HC. (**B**). Heat map of total IgG proteomics showing the top 50 proteins in each group. The upregulated IgG antibodies in GBM, HC, and Met compared to MMA are highlighted. (**C**,**D**). Proteomic analysis of plasma EVs demonstrated the unique proteins in GBM EVs. (**C**). PCA shows clear clusters of proteins in each EV group. (**D**). Heat map showing unique proteins detected in GBM EVs including immunoglobulin heavy and light chains and apolipoproteins.

We performed proteomic analysis via mass spectrometry of brain tumor plasma IgG and plasma-derived EVs. For total IgG proteomics, we did not observe clear distinctions of proteins among the 4 groups of samples. Principal Component Analysis (PCA) showed distinctions among the four groups of samples including GBM, MMA, metastatic brain tumors (Met), and HC (Figure 5A). The heat map shows upregulated IgG antibodies in GBM, HC, and Met compared to MMA (Figure 5B). However, clearer clusters of proteins in each group were present in EV proteomics (Figure 5C), showing unique proteins detected in GBM EVs including immunoglobulin heavy and light chains and apolipoproteins (Figure 5D).

**Figure 6:**
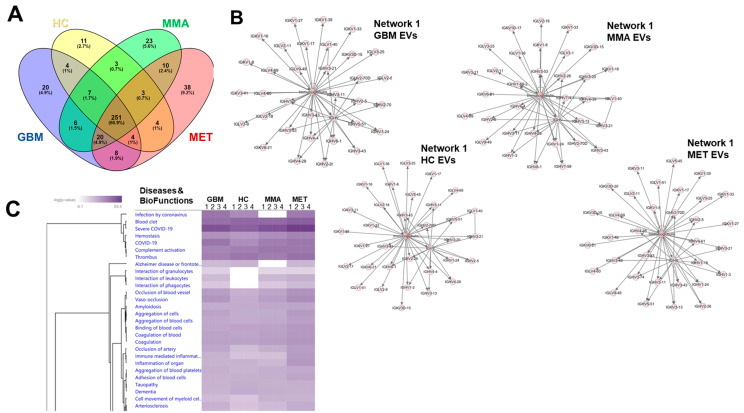
Venn diagram Ingenuity Pathway Analysis (IPA) readouts of plasma EV proteomes. (**A**). Venn diagram showing overlapping and unique protein numbers from each plasma EV category. (**B**). Top networks derived from IPA Core Analysis for each plasma EV proteome showing the predominance of immunoglobulin networks (Networks were all entitled “Cancer, Infectious Disease, Organismal Injury and Abnormalities”; scores = 65–67; 33 focus molecules). (**C**). Comparison Analysis conducted on all four plasma EV datasets from four different patients or controls showing hierarchical clustering of the top 30 Disease and BioFunction significant categories. This is a partial representation; the full table of all 636 categories is found in Table S5.

Substantial overlap was present among overall protein identities across groups (Figure 6A), but with evident differences. Ingenuity Pathway Analysis (IPA) Core Analyses of total proteins in each of the groups (Table S5) found abundant immunoglobulins in the top networks for each group (Figure 6B), as suggested in Figure 5D. IPA Comparison Analysis, Diseases and BioFunctions in particular, highlights differences between the cancer plasma EV proteomes and the HC plasma EV proteome noting the lack of interactions of granulocytes, lymphocytes, and phagocytes as a category in the HC EV proteome based on hierarchical clustering (Figure 6C).

**Figure 7:**
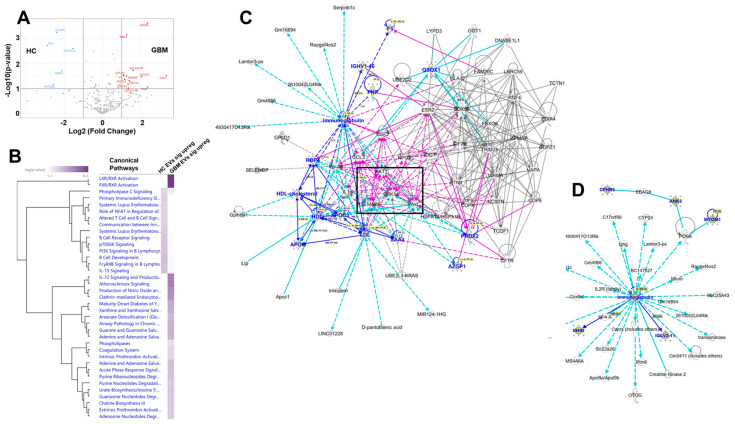
Differential protein content between HC and GBM plasma EV proteomes, and IPA readouts using only those proteins. (**A**). Volcano graph showing significantly different protein content comparing HC plasma EVs (left side, blue designations) and GBM plasma EVs (right side, red designations. (**B**). Comparison analysis of the significantly different proteins (HC plasma EV proteins left column; GBM plasma EV proteins right column). Shown are all the significant canonical pathways rendered in hierarchical clustering. (**C**). Merge of the two interactomes from network analysis of significantly expressed GBM plasma EV proteins; black box surrounds signaling nodes of AKT, ERK1/2, and p38MAPK. Network names: (1) “Lipid Metabolism, Molecular Transport, Small Molecule Biochemistry”; score = 33; 11 focus molecules; (2) “Infectious Diseases, Organismal Injury and Abnormalities, Protein Synthesis”; score = 4; 2 focus molecules. (**D**). Sole network from significantly expressed HC plasma EV proteins. Network name: “Auditory Disease, Hereditary Disorder, Neurologic Disease”; score = 15; 5 focus molecules. For (**C**,**D**), proteins identified in the datasets are listed in blue color.

A deeper analysis of the differentially expressed proteins found when comparing HC plasma EVs vs. GBM patient plasma EVs (Figure 7A) shows that, despite the relatively small numbers of proteins on input, hierarchical clustering of the comparative canonical pathways finds significant differences in many pathways (Figure 7B). Network interactomes reveal cancer signaling pathway nodes for the over-represented proteins in the plasma EVs of GBM patients, with another immunoglobulin-centered network for HC EV plasma EVs (Figure 7C,D). These results suggest that immunoglobulins associated with plasma EVs from patients with GBM may play important roles in EV activities, and the differentially expressed EV proteins tie to prominent cancer signaling pathways (AKT1; p38MAPK; ERK1/2).

In the Supplementary Files, Volcano plots for comparing up- and down-regulated proteins in GBM and HC purified IgG and EV samples are presented in Figures S1 and S2, respectively. The patient demographics are presented in Table S1. The protein concentrations of samples used for Mass Spectrometry are shown in Table S2. The proteomic results for EV and IgG are listed in Tables S3 and S4, respectively. The complete list of IPA comparison analysis of all 4 types of EV samples is available in Table S5.

## 4. Discussion

We demonstrated the unique phenotype and cytotoxic functions of GBM extracellular vesicles: (1) GBM EVs are smaller, contain unique protein profiles, and have no relationship between EV size and number; (2) GBM plasma contains highly elevated levels of IgG Fc antibodies; (3) GBM EVs induce IgG-mediated, complement-dependent cytotoxicity in neurons.

GBM cells communicate with surrounding cells by releasing EVs containing proteins, lipids, and RNA [19], suggesting that GBM EVs could play a critical role in tumor pathogenesis. We characterized GBM plasma EV size and number utilizing nanoparticle tracking analysis (NTA) and protein concentration by BCA assay. We showed that GBM EVs have no relationship between size and particle number, but that there is a strong correlation in HC and MMA EVs, indicating the uniqueness of GBM plasma EVs. Proteomics analysis of GBM EVs revealed that elevated proteins for EVs and plasma included immunoglobulin heavy and light chains, as well as apolipoproteins, further supporting the connection between GBM EVs and plasma. These findings also relate to GBM plasma EV-induced neurotoxicity. The significance of these observations requires further investigation.

Using multiple capture ELISA, we demonstrated a highly elevated level of IgG Fc antibodies in GBM plasma compared to MMA and HC; however, while we did not observe an increased IgG in GBM EVs utilizing this approach, a similar trend exists. Interestingly, using the Fc-III peptides capture reagent, we showed the most distinct differences in IgG levels amongst GBM, MMA, and HC. We included over 80 plasma samples for each group and repeated the experiments at least once.

GBM is a complex brain cancer with unknown etiology. Recent genetic profiling studies of tumor tissues have found somatic mutations in genes such as the TERT, PTEN, and epidermal growth factor receptor (EGFR) [40,41]. These genetic alternations, along with mutations in tumor suppressor TP53 and related pathway alterations [42,43], could initiate tumor pathogenesis. Tumor cells are frequently adapted to evade the body’s immune surveillance by secreting various cytokines, growth factors, chemokines, and other effector molecules, often in the context of EVs, consequently shaping an immunosuppressive tumor microenvironment [44,45,46,47,48]. Altered plasma IgG levels in GBM patients vs. HC could indicate immune modulation (immunosuppression or inflammation). Our study did not find any significant differences in plasma total IgG levels between GBM and HC, which is consistent with antibody responses against glioma antigens (naturally or induced in GBM patients [49,50] However, we found that GBM IgG had a higher binding capability to Fc peptide than HC IgG, indicating possible structural changes in GBM IgG vs. HC IgG that alter epitope availability, such as glycosylation or other post-translational modifications.

Because of the elevated levels of GBM plasma IgG Fc and IgG1, we wondered if GBM EVs may induce cytotoxicity in neuronal cells. IgG molecules can trigger pro-inflammatory responses mediated by their fragment crystallizable domain (Fc). Activation of the classical complement pathway via C1q binding to human IgG1 and IgG3 can result in cell lysis [51]. Using the neuroblastoma cell line SH-SY5Y as a neuronal model system [52], we evaluated GBM EV-induced neuronal cytotoxicity using multiple EV purification methods from both plasma and tumor cell lines. We applied GBM EVs purified by ExoEasy, ExoQuick, and ultracentrifugation methods, onto neuroblastoma cells, primary human neurons, and mouse brain slices. The neuronal cytotoxicity was evaluated by LIVE/DEAD™ Viability/Cytotoxicity kits, propidium Iodide, and RealTime-Glo Annexin V Apoptosis/Necrosis kits. We demonstrated regardless of EV source (plasma or tumor cell line), isolation method, or detection assay, GBM EVs produced complement-dependent cytotoxicity in a dose- and time-dependent manner. The significance of the complement dependency for GBM EV neuronal cytotoxicity was supported by data showing that higher levels of cytotoxicity were produced when normal human serum (as complement source) was included (Figure 3D,E), and blocking complement component C1q with anti-C1q antibodies significantly reduced GBM EV induced cytotoxicity (Figure 3F).

We applied the highly specific IgG-degrading enzyme IdeS to demonstrate the importance of intact IgG in GBM EV-induced cytotoxicity. We showed that GBM plasma EVs treated with IdeS induced significantly lower levels of neuronal cell death compared to untreated EVs (Figure 3B), suggesting the significance of IgG involvement in GBM EV neuron killing.

However, there are differences between GBM plasma and tumor cell line EVs. For example, significantly lower levels of cytotoxicity were induced by IdeS-digested GBM pooled plasma EV (ppEV)-treated cells compared to undigested EVs (*p* = 0.0031). However, no difference was seen in IdeS-digested GBM cell line-derived EV (mEV)-treated cells compared to undigested EVs, indicating that the IgG antibodies on the surface of GBM media EVs may not be exposed for IdeS digestion, or there are IgG-independent cell killing mechanisms for GBM cell line EVs. The highly significant reduction of cytotoxicity after blocking of C1q on GBM EVs indicates that complement components on the GBM EV surface are critical.

We isolated EVs from plasma using a polyethylene glycol-based method (ExoQuick, Systems Biosciences, Cat# EXOQ20A-1, Palo Alto, CA, USA), followed by measures of Fc-bound IgG in GBM, MMA, and HC EV utilizing an ELISA-based assay. There were no significant differences in IgG levels among GBM, MMA, and HC EV. Since we found that GBM EVs can cause higher neurotoxicity as compared to HC EVs, regardless of the EV purification method, it suggests that GBM EVs may contain some unknown factor that contributes to neurotoxicity as compared to HC EVs.

GBM is characterized by progressive neurological deficits. However, whether these symptoms result from direct or indirect damage to neurons is still unresolved. A recent study in a *Drosophila* model demonstrates that GBM cells “vampirize” WNT/*wg* from neurons by surrounding the neurons with microtubule-based cell extensions. This triggers a JNK/MMP signaling loop allowing GBM cell migration and subsequent neurodegeneration [53]. Our study here showing that GBM EVs induce complement-dependent cytotoxicity in neurons provides a foundation for further investigation of the mechanism of neurodegeneration in GBM.

In this study, we found that GBM EVs could induce higher neurotoxicity than EVs isolated from HC or other brain tumors. The molecular nature of this GBM EV-induced cytotoxicity is still unknown. Our preliminary results suggest that surface IgG or IgG-like molecules in/on the EVs could play a role, as IdeS digestion, which breaks down IgG protein, diminishes EV-driven cytotoxicity. Another possible pathway could be mediated by IgG-like or other unknown molecules released by EV cargo, as proteins could be packaged as EV cargo and released at the surface or inside the target cells. The implication for GBM EV’s cytotoxicity for tumor progression or cancer, in general, was unknown. Future experiments are needed to test if these EVs could aid tumor progression.

## 5. Conclusions

In this study, we showed that GBM EVs possess unique characteristics, inducing IgG-mediated, complement-dependent, cytotoxicity in neurons. Our study is limited by the small sample size for GBM EVs, and limited assay formats. Further investigations are needed to reveal the mechanism of GBM EV-induced neuronal death.

## Data Availability

All data are available for review and materials are available upon reasonable request.

## Supplementary Materials

The following supporting information can be downloaded at: https://www.mdpi.com/2227-9059/10/11/2718/s1, Figure S1: Volcano plot of GBM and HC purified IgG proteomics; Figure S2: Volcano plot of GBM and HC purified EV proteomics; Table S1: Patient Demographics; Table S2: Protein concentrations of samples for Mass Spectrometry, Table S3: Proteomic results of EV samples; Table S4: Proteomic results of IgG samples; Table S5: IPA comparison analysis all 4 EV samples full table.

## References

[1] Wirsching H.-G., Galanis E., Weller M., Berger M.S., Weller M. (2016). Chapter 23-Glioblastoma. Handbook of Clinical Neurology.

[2] Lukas R.V., Wainwright D.A., Ladomersky E., Sachdev S., Sonabend A.M., Stupp R. (2019). Newly Diagnosed Glioblastoma: A Review on Clinical Management. Oncology (Williston Park N. Y.).

[3] Ostrom Q.T., Cioffi G., Waite K., Kruchko C., Barnholtz-Sloan J.S. (2021). CBTRUS Statistical Report: Primary Brain and Other Central Nervous System Tumors Diagnosed in the United States in 2014-2018. Neuro Oncol..

[4] Tamimi A.F., Juweid M. (2017). Epidemiology and Outcome of Glioblastoma. Glioblastoma.

[5] Liu E.K., Sulman E.P., Wen P.Y., Kurz S.C. (2020). Novel Therapies for Glioblastoma. Curr. Neurol. Neurosci. Rep..

[6] Poon M.T.C., Sudlow C.L.M., Figueroa J.D., Brennan P.M. (2020). Longer-term (≥2 years) survival in patients with glioblastoma in population-based studies pre- and post-2005: A systematic review and meta-analysis. Sci. Rep..

[7] Huntoon K., Toland A.M.S., Dahiya S. (2020). Meningioma: A Review of Clinicopathological and Molecular Aspects. Front. Oncol..

[8] Patra D.P., Savardekar A.R., Dossani R.H., Narayan V., Mohammed N., Nanda A. (2018). Meningioma: The Tumor That Taught Us Neurosurgery. World Neurosurg..

[9] Preusser M., Brastianos P.K., Mawrin C. (2018). Advances in meningioma genetics: Novel therapeutic opportunities. Nat. Rev. Neurol..

[10] Jung C.S., Foerch C., Schänzer A., Heck A., Plate K.H., Seifert V., Steinmetz H., Raabe A., Sitzer M. (2007). Serum GFAP is a diagnostic marker for glioblastoma multiforme. Brain.

[11] Kiviniemi A., Gardberg M., Frantzén J., Parkkola R., Vuorinen V., Pesola M., Minn H. (2015). Serum levels of GFAP and EGFR in primary and recurrent high-grade gliomas: Correlation to tumor volume, molecular markers, and progression-free survival. J. Neurooncol..

[12] Tichy J., Spechtmeyer S., Mittelbronn M., Hattingen E., Rieger J., Senft C., Foerch C. (2016). Prospective evaluation of serum glial fibrillary acidic protein (GFAP) as a diagnostic marker for glioblastoma. J. Neurooncol..

[13] Dong L., Li Y., Han C., Wang X., She L., Zhang H. (2014). miRNA microarray reveals specific expression in the peripheral blood of glioblastoma patients. Int. J. Oncol..

[14] Tang H., Liu Q., Liu X., Ye F., Xie X., Xie X., Wu M. (2015). Plasma miR-185 as a predictive biomarker for prognosis of malignant glioma. J. Cancer Res. Ther..

[15] Lai N.S., Wu D.G., Fang X.G., Lin Y.C., Chen S.S., Li Z.B., Xu S.S. (2015). Serum microRNA-210 as a potential noninvasive biomarker for the diagnosis and prognosis of glioma. Br. J. Cancer.

[16] Graner M.W. (2018). Extracellular vesicles in cancer immune responses: Roles of purinergic receptors. Semin. Immunopathol..

[17] Pegtel D.M., Gould S.J. (2019). Exosomes. Annu. Rev. Biochem..

[18] Harding C.V., Heuser J.E., Stahl P.D. (2013). Exosomes: Looking back three decades and into the future. J. Cell Biol..

[19] Maas S.L.N., Breakefield X.O., Weaver A.M. (2017). Extracellular Vesicles: Unique Intercellular Delivery Vehicles. Trends Cell Biol..

[20] Saint-Pol J., Gosselet F., Duban-Deweer S., Pottiez G., Karamanos Y. (2020). Targeting and Crossing the Blood-Brain Barrier with Extracellular Vesicles. Cells.

[21] Hong S.B., Yang H., Manaenko A., Lu J., Mei Q., Hu Q. (2019). Potential of Exosomes for the Treatment of Stroke. Cell Transplant..

[22] Yaghoubi Y., Movassaghpour A., Zamani M., Talebi M., Mehdizadeh A., Yousefi M. (2019). Human umbilical cord mesenchymal stem cells derived-exosomes in diseases treatment. Life Sci..

[23] Kalluri R., LeBleu V.S. (2020). The biology, function, and biomedical applications of exosomes. Science.

[24] Graner M.W. (2019). Roles of Extracellular Vesicles in High-Grade Gliomas: Tiny Particles with Outsized Influence. Annu. Rev. Genom. Hum. Genet..

[25] Garzon-Muvdi T., Bailey D.D., Pernik M.N., Pan E. (2020). Basis for Immunotherapy for Treatment of Meningiomas. Front. Neurol..

[26] Nieland L., Morsett L.M., Broekman M.L.D., Breakefield X.O., Abels E.R. (2021). Extracellular Vesicle-Mediated Bilateral Communication between Glioblastoma and Astrocytes. Trends Neurosci..

[27] Arneth B. (2019). Tumor Microenvironment. Medicina.

[28] Müller Bark J., Kulasinghe A., Chua B., Day B.W., Punyadeera C. (2020). Circulating biomarkers in patients with glioblastoma. Br. J. Cancer.

[29] Mahaley M.S., Brooks W.H., Roszman T.L., Bigner D.D., Dudka L., Richardson S. (1977). Immunobiology of primary intracranial tumors. Part 1: Studies of the cellular and humoral general immune competence of brain-tumor patients. J. Neurosurg..

[30] Wilcox J.A., Ramakrishna R., Magge R. (2018). Immunotherapy in Glioblastoma. World Neurosurg..

[31] Le Rhun E., Preusser M., Roth P., Reardon D.A., van den Bent M., Wen P., Reifenberger G., Weller M. (2019). Molecular targeted therapy of glioblastoma. Cancer Treat. Rev..

[32] Desai K., Hubben A., Ahluwalia M. (2019). The Role of Checkpoint Inhibitors in Glioblastoma. Target. Oncol..

[33] Al-Rashed M., Foshay K., Abedalthagafi M. (2019). Recent Advances in Meningioma Immunogenetics. Front. Oncol..

[34] Capello M., Vykoukal J.V., Katayama H., Bantis L.E., Wang H., Kundnani D.L., Aguilar-Bonavides C., Aguilar M., Tripathi S.C., Dhillon D.S. (2019). Exosomes harbor B cell targets in pancreatic adenocarcinoma and exert decoy function against complement-mediated cytotoxicity. Nat. Commun..

[35] Yu X., Zizzo Z., Kennedy P.G. (2021). An appraisal of antigen identification and IgG effector functions driving host immune responses in multiple sclerosis. Mult. Scler. Relat. Disord..

[36] Zhou W., Craft J., Ojemann A., Bergen L., Graner A., Gonzales A., He Q., Kopper T., Smith M., Graner M.W. (2022). Glioblastoma Extracellular Vesicle-Specific Peptides Inhibit EV-Induced Neuronal Cytotoxicity. Int. J. Mol. Sci..

[37] Oushy S., Hellwinkel J.E., Wang M., Nguyen G.J., Gunaydin D., Harland T.A., Anchordoquy T.J., Graner M.W. (2018). Glioblastoma multiforme-derived extracellular vesicles drive normal astrocytes towards a tumour-enhancing phenotype. Philos. Trans. R. Soc. B Biol. Sci..

[38] DeLano W.L., Ultsch M.H., de Vos A.M., Wells J.A. (2000). Convergent solutions to binding at a protein-protein interface. Science.

[39] Collin M., Björck L. (2017). Toward Clinical use of the IgG Specific Enzymes IdeS and EndoS against Antibody-Mediated Diseases. Methods Mol. Biol..

[40] Brennan C.W., Verhaak R.G., McKenna A., Campos B., Noushmehr H., Salama S.R., Zheng S., Chakravarty D., Sanborn J.Z., Berman S.H. (2013). The somatic genomic landscape of glioblastoma. Cell.

[41] Fujimoto K., Arita H., Satomi K., Yamasaki K., Matsushita Y., Nakamura T., Miyakita Y., Umehara T., Kobayashi K., Tamura K. (2021). TERT promoter mutation status is necessary and sufficient to diagnose IDH-wildtype diffuse astrocytic glioma with molecular features of glioblastoma. Acta Neuropathol..

[42] Masui K., Cloughesy T.F., Mischel P.S. (2012). Review: Molecular pathology in adult high-grade gliomas: From molecular diagnostics to target therapies. Neuropathol. Appl. Neurobiol..

[43] Zhang Y., Dube C., Gibert M., Cruickshanks N., Wang B., Coughlan M., Yang Y., Setiady I., Deveau C., Saoud K. (2018). The p53 Pathway in Glioblastoma. Cancers.

[44] Dapash M., Hou D., Castro B., Lee-Chang C., Lesniak M.S. (2021). The Interplay between Glioblastoma and Its Microenvironment. Cells.

[45] DeCordova S., Shastri A., Tsolaki A.G., Yasmin H., Klein L., Singh S.K., Kishore U. (2020). Molecular Heterogeneity and Immunosuppressive Microenvironment in Glioblastoma. Front. Immunol..

[46] Desland F.A., Hormigo A. (2020). The CNS and the Brain Tumor Microenvironment: Implications for Glioblastoma Immunotherapy. Int. J. Mol. Sci..

[47] Tomaszewski W., Sanchez-Perez L., Gajewski T.F., Sampson J.H. (2019). Brain Tumor Microenvironment and Host State: Implications for Immunotherapy. Clin. Cancer Res..

[48] Hellwinkel J.E., Redzic J.S., Harland T.A., Gunaydin D., Anchordoquy T.J., Graner M.W. (2016). Glioma-derived extracellular vesicles selectively suppress immune responses. Neuro Oncol..

[49] He S.J., Gu Y.Y., Yu L., Luo B., Fan R., Lin W.Z., Lan X.W., Lin Y.D., Zhang Q.M., Xiao S.W. (2014). High expression and frequently humoral immune response of melanoma-associated antigen D4 in glioma. Int. J. Clin. Exp. Pathol..

[50] Choi B.D., Archer G.E., Mitchell D.A., Heimberger A.B., McLendon R.E., Bigner D.D., Sampson J.H. (2009). EGFRvIII-targeted vaccination therapy of malignant glioma. Brain Pathol..

[51] Quast I., Keller C.W., Maurer M.A., Giddens J.P., Tackenberg B., Wang L.X., Münz C., Nimmerjahn F., Dalakas M.C., Lünemann J.D. (2015). Sialylation of IgG Fc domain impairs complement-dependent cytotoxicity. J. Clin. Investig..

[52] Forster J.I., Köglsberger S., Trefois C., Boyd O., Baumuratov A.S., Buck L., Balling R., Antony P.M. (2016). Characterization of Differentiated SH-SY5Y as Neuronal Screening Model Reveals Increased Oxidative Vulnerability. J. Biomol. Screen.

[53] Portela M., Venkataramani V., Fahey-Lozano N., Seco E., Losada-Perez M., Winkler F., Casas-Tintó S. (2019). Glioblastoma cells vampirize WNT from neurons and trigger a JNK/MMP signaling loop that enhances glioblastoma progression and neurodegeneration. PLoS Biol..

