# Supplementary material for: Phenotype and Neuronal Cytotoxic Function of Glioblastoma Extracellular Vesicles
Source: Biomedicines. 2022 Oct 27;10(11):2718. doi: 10.3390/biomedicines10112718 (PMC9688005; doi:10.3390/biomedicines10112718)
Supplement: Supplementary file 1 [file biomedicines-10-02718-s001.zip › biomedicines-1930407 Table S1 Patient Demographics.pdf]

Table S1. Patient Demographics.

	GBM	MMA	Controls
Subject (n)	82	83	50
Male (n)	55	26	23
Female (n)	27	57	27
Age (years, mean ± SD)	60.4±11.3	56.9±12.0	45.1±13.1
Diagnostic	Glioblastoma, WHO grade IV	Meningioma, WHO grade I	Healthy Controls (10M/18F) Other non-tumor
