# Supplementary material for: Phenotype and Neuronal Cytotoxic Function of Glioblastoma Extracellular Vesicles
Source: Biomedicines. 2022 Oct 27;10(11):2718. doi: 10.3390/biomedicines10112718 (PMC9688005; doi:10.3390/biomedicines10112718)
Supplement: Supplementary file 1 [file biomedicines-1930407 Supplementary Figures.pdf › biomedicines-1930407 Supplementary Figures.pdf]

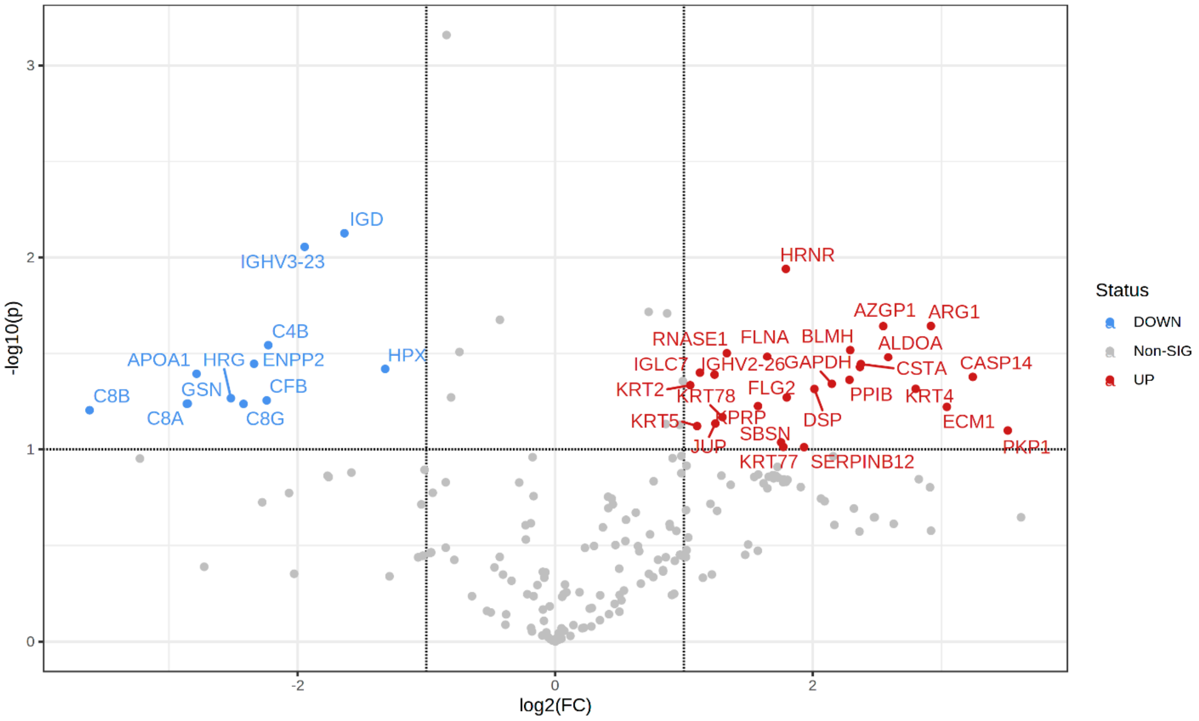

Figure S1. Volcano plot of GBM vs HC purified IgG proteomics. Up: proteins that are increased in GBM purified IgG vs HC purified IgG, Down: proteins that are decreased in GBM purified IgG vs HC purified IgG, Non-sig: proteins that not significantly changed between GBM and HC purified IgG

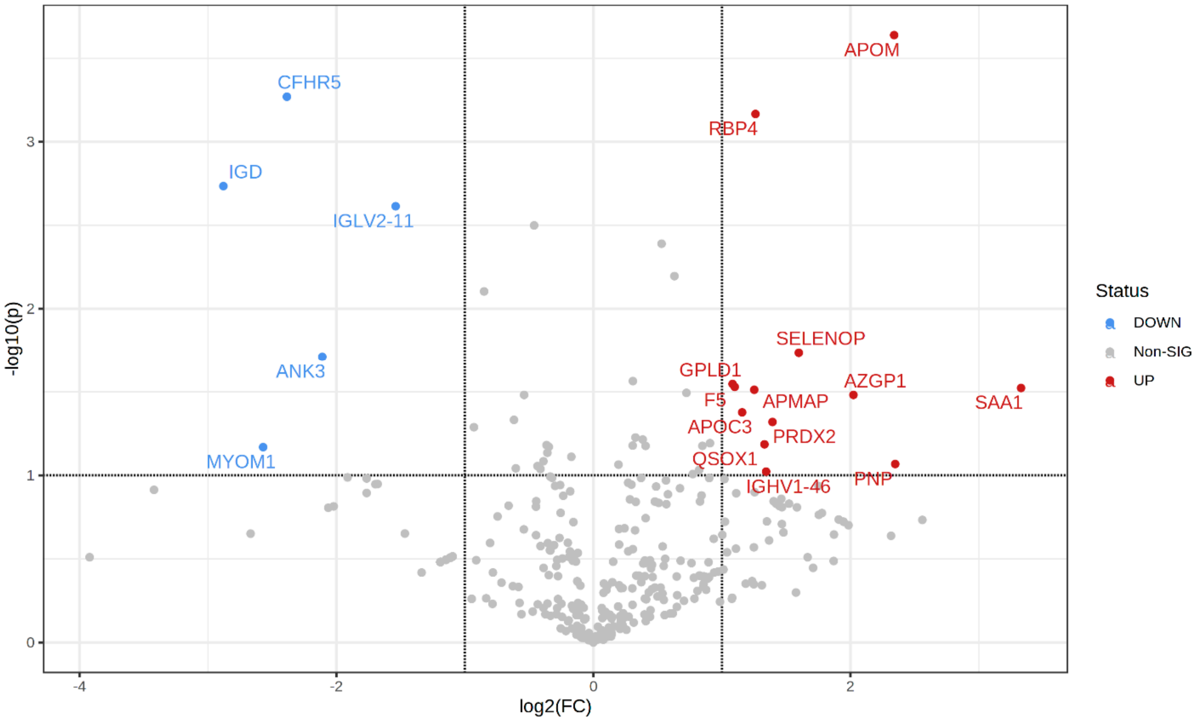

Figure S2. Volcano plot of GBM vs HC EV proteomics. Up: proteins that are increased in GBM EV vs HC EV, Down: proteins that are decreased in GBM EV vs HC EV, Non-sig: proteins that not significantly changed between GBM and HC EV.
